# Should sex-ratio distorting parasites abandon horizontal transmission?

**DOI:** 10.1186/1471-2148-11-370

**Published:** 2011-12-21

**Authors:** Joseph E Ironside, Judith E Smith, Melanie J Hatcher, Alison M Dunn

**Affiliations:** 1 Institute of Biological, Environmental and Rural Sciences, Aberystwyth University, Aberystwyth, Ceredigion SY23 3DA, UK; 2 University of Salford, Salford, Greater Manchester M5 4WT, UK; 3 School of Biological Sciences, University of Bristol, Woodland Road, Bristol BS8 1UG, UK; 4 Institute of Integrative and Comparative Biology, Faculty of Biological Science, University of Leeds, Leeds LS2 9JT, UK

## Abstract

**Background:**

Sex-ratio distorting parasites are of interest due to their effects upon host population dynamics and their potential to influence the evolution of host sex determination systems. In theory, the ability to distort host sex-ratios allows a parasite with efficient vertical (hereditary) transmission to dispense completely with horizontal (infectious) transmission. However, recent empirical studies indicate that some sex-ratio distorting parasites have retained the capability for horizontal transmission.

**Results:**

Numerical simulations using biologically realistic parameters suggest that a feminising parasite is only likely to lose the capability for horizontal transmission if its host occurs at low density and/or has a male-biased primary sex ratio. It is also demonstrated that even a small amount of horizontal transmission can allow multiple feminising parasites to coexist within a single host population. Finally it is shown that, by boosting its host's rate of population growth, a feminising parasite can increase its own horizontal transmission and allow the invasion of other, more virulent parasites.

**Conclusions:**

The prediction that sex-ratio distorting parasites are likely to retain a degree of horizontal transmission has important implications for the epidemiology and host-parasite interactions of these organisms. It may also explain the frequent co-occurrence of several sex-ratio distorting parasite species in nature.

## Background

Sex-ratio distorting (SRD) parasites are able to enhance their transovarial vertical transmission from infected females to their offspring by causing infected hosts to produce more female offspring than do uninfected individuals. This is accomplished either through direct feminisation of genetically male embryos or by killing male embryos and hence increasing the fitness of their infected female siblings by eliminating intra-brood competition. SRD is widespread among parasitic bacteria, including *Wolbachia *[1], rickettsias [2], spiroplasmas [3,4], flavobacteria [5] and 'Bacteroidetes' [6]. Eukaryotic SRD parasites also occur within the Microspora [7] and Haplospora [8]. The majority of SRD parasites possess very efficient mechanisms of transovarial vertical transmission and do not normally appear to undergo horizontal transmission. This observation has been explained by the theoretical prediction that sex-ratio distortion enables a parasite to maintain its prevalence through vertical transmission alone [9,10]. Given the inherent trade-off between horizontal transmission, with its demand for host resources, and vertical transmission which is limited by the host's reproductive success [11-13], there are reasonable grounds to propose that SRD parasites are selected to abandon horizontal transmission entirely.

Although horizontal transmission is not essential to the survival of an SRD parasite in theory, there is mounting evidence that it occurs in nature, suggesting some evolutionary benefit. Lack of congruence between the phylogenies of host and SRD parasite genes suggests a degree of horizontal transmission, both between and within host species [14-16]. The high incidence of hosts infected with multiple strains of SRD parasites in some populations also suggests that parasites can be reacquired through horizontal transmission [15,17] as does the presence of very similar parasite strains in distantly related host species [18,19]. SRD strains of the bacterium *Wolbachia *also demonstrate recombination between strains [20] and do not appear to suffer from the genomic degeneration characteristic of vertically transmitted mutualistic endosymbionts [21,22].

A growing body of direct empirical evidence also indicates that some bacterial SRD parasites are capable of horizontal transmission. Horizontal transmission occurs under natural conditions in the male-killing SRD bacterium *Arsenophonus nasoniae*, a parasite that is transmitted horizontally between larvae of its parasitoid host *Nasonia vitripennis *when multiple females lay their eggs in the same insect pupa [23,24]. Parthenogenesis-inducing *Wolbachia *also undergo natural horizontal transmission between larval *Trichogramma *parasitoids feeding within the same butterfly egg [25] and feminising *Wolbachia *can be transmitted through blood-blood contact between injured *Armadillidium vulgare *hosts [26]. Male-killing SRD *Spiroplasma *can infect *Drosophila willistoni *that are fed upon the tissue of infected individuals [27] and can also be transmitted between *Drosophila *hosts by ectoparasitic mites [28]. Finally, artificial infections of insect and crustacean hosts with SRD *Wolbachia *[29,30] can be performed in the laboratory by injecting infected tissue into the body cavity of uninfected individuals, suggesting that horizontal transmission may occur under natural conditions through blood-blood contact.

Among microsporidia, the putative SRD species *Pleistophora mulleri *[7] produces heavy infections of muscle tissue with significant pathogenic effects [31] and is horizontally transmitted through cannibalism [32]. The feminising microsporidia *Dictyocoela duebenum *and *D. muelleri *also produce heavy infections of muscle tissue in their *Gammarus *hosts, indicating probable horizontal transmission [33]. Although the feminising microsporidian *Nosema granulosis *produces only light infections, it can be artificially transmitted between *Gammarus duebeni *hosts through injection of infected host tissue [34]. Both *D. duebenum *and *N. granulosis *have been discovered in recently established European populations of the Ponto-Caspian invader *Dikerogammarus villosus *but appear to be absent from *D. villosus *in its native range [35]. This suggests that horizontal transmission of feminising microsporidia to *D. villosus *from native European hosts has occurred on an ecological timescale.

It is argued that an SRD microorganism is likely to maintain a capability for effective horizontal transmission if it is capable of persisting outside of the host, maintains a broad range of metabolic functions, is capable of modulating the host immune system and is able to invade through host epithelia [24]. Among SRD bacteria, *Arsenophonus nasoniae *retains these traits while the more specialised endosymbiont *Wolbachia *does not. All known SRD microsporidia produce spores with polar filaments [7,36,37], indicating the ability to persist outside of the host and to invade new host cells. Furthermore, these SRD microsporidia all appear to have evolved recently from horizontally transmitted non-SRD ancestors [7,37,38]. The feminising haplosporidian *Marteilia *sp. also displays evidence of sporogenesis, can exist outside of host cells and appears to be able to move between organs by digesting the basal membrane [8].

An important consequence of the ability of parasites to spread by both vertical transmission and horizontal transmission is that the prevalence of different parasite species or strains can be limited by different factors. This creates the potential for several competing SRD parasites to coexist within a single host population [39,40]. In contrast, SRD parasites that lack horizontal transmission should never be able to coexist in a panmictic population of susceptible hosts because the parasite species or strain with the most efficient vertical transmission should always displace its competitors [41-45]. Coexistence of SRD parasites occurs frequently in natural host populations [3,42,46] and previous theoretical attempts to explain coexistence in the absence of horizontal transmission have employed complex mechanisms involving polymorphism for host resistance genes and/or parasite virulence genes [43], sometimes in combination with spatial population structure [41].

More recent theoretical studies have analysed models including SRD parasites capable of both vertical and horizontal transmission. Engelstadter and Hurst [47] consider a male-killing parasite that is capable of sexual transmission from males to females while Yamauchi et al [48] consider the case of a feminising parasite with some paternal transmission. In both cases, horizontal transmission occurs only through adult males and so SRD and horizontal transmission are mutually exclusive. Both studies conclude that the trade-off between SRD and horizontal transmission can result in the coexistence of multiple parasite strains with different levels of SRD. However, these models are unsuitable for addressing the question of whether SRD can prevent the invasion of horizontal transmission because neither model attributes any fitness cost to horizontal transmission specifically.

Jones et al [49] consider a vertically transmitted feminising parasite that co-infects with a parasite capable of direct horizontal transmission. In this case, horizontal transmission occurs through females and so SRD and horizontal transmission are not mutually exclusive. A fitness cost, in terms of virulence, is also attributed to horizontal transmission. This study draws the conclusion that a vertically transmitted feminising parasite can select for increased virulence in a co-infecting horizontally transmitted parasite. However, the model does not consider the case of a parasite capable of both vertical and horizontal transmission and also ignores horizontal parasite transmission through male hosts. It is therefore unsuitable for investigating tradeoffs between horizontal transmission and feminisation.

The empirical examples given above suggest that under certain conditions the capability for horizontal transmission may have been retained or re-evolved by SRD parasites. In most documented cases, horizontal transmission of SRD parasites occurs through direct contact between infected and uninfected hosts (cannibalism or blood-blood contact), suggesting that horizontal transmission is a density-dependent process. Models assuming frequency-dependent forms of horizontal transmission, such as sexual transmission or paternal inheritance, are more tractable. However, such models lack biological realism in the case of SRDs since, to the authors' knowledge, there are no documented cases of sexual transmission or paternal inheritance in feminising or male-killing parasites. Given that horizontal transmission via cannibalism or blood-blood contact can occur via male or female hosts, a biologically realistic model must also consider transmission through both sexes. The production of analytically tractable models typically also requires assumptions such as complete efficiency of feminisation or the production of a 1:1 sex ratio by uninfected hosts. Such assumptions are demonstrably invalid for many of the systems in which sex-ratio distortion actually occurs. In the well-studied case of the microsporidian parasite *Nosema granulosis*, for example, the sex-ratio of uninfected *Gammarus duebeni *hosts is biased [42] and only a proportion of infected embryos are feminised [50]. Given that the effectiveness of feminisation as a means of enhancing vertical transmission depends crucially upon the host sex ratio and efficiency of feminisation, reducing these parameters to constants clearly limits the applicability of a model to real host-parasite systems.

In this theoretical study we produce a general model of a host-parasite system in which parasites are capable of vertical transmission, feminisation and horizontal transmission via direct contact between infected and uninfected hosts. Simulation, with biologically realistic parameters, is used to examine the effects of varying host and parasite parameters upon the ability of parasites to invade and persist within host populations.

## Methods

### The Model

In order to examine parasite and host population dynamics when both vertical and horizontal transmission occur, we combined the standard parasitological approach to modeling horizontal transmission developed by Anderson & May [51] with the classical population genetic approach commonly applied in models of vertical transmission [45,52]. The model follows the basic form of models produced by Anderson & May (1981) except that the host population is assumed to be dioecious and its birth rate is a function of the number of females. The division of the host population into males and females allows the effects of a feminising parasite upon the population sex ratio to be included in the model. Equations were derived and examined in Maple (^®^2002 Waterloo Maple Inc.). A list of parameters is provided in Table 1.

**Table 1:** List of parameters used in models with definitions

Parameter	Definition
*a*	Birth rate
*g*	Uninfected sex ratio (proportion of female offspring)
*b*_0_	Basal death rate
*s*	Density dependent mortality (constant coefficient of *H*)
*t*	Time required for one host generation
*ε*	Efficiency of vertical transmission
*θ*	Efficiency of feminisation
*α*	Virulence (viability cost)
*γ*	Rate at which host clears parasite
*λ*	Component of virulence due to vertical transmission (constant coefficient of *ε*)
*μ*	Efficiency of superinfection
*β*	Rate of horizontal transmission (proportionality constant)
*σ*	Component of virulence due to horizontal transmission (constant coefficient of *β*)
*H*	Total number of hosts in population
*X_m_*	Number of uninfected male hosts
*X_f_*	Number of uninfected female hosts
*Y_m_*	Number of male hosts infected with parasite 1
*Y_f_*	Number of female hosts infected with parasite 1
*Z_m_*	Number of male hosts infected parasite 2
*Z_f_*	Number of female hosts infected with parasite 2
*K*	Carrying capacity
H*	Equilibrium population size
Y*	Equilibrium number of hosts infected with parasite 1
Z*	Equilibrium number of hosts infected with parasite 2
*y*	Prevalence of parasite 1
y*	Equilibrium prevalence of parasite 1
*z*	Prevalence of parasite 2
z*	Equilibrium prevalence of parasite 2
*R*_0_	Basic reproductive rate of uninfected cytotype in uninfected population
*R*_1_	Basic reproductive rate of parasite 1
*R*_2_	Basic reproductive rate of parasite 2

A population of *H *hosts contains *X_m _*males and *X_f _*females. The birth rate of the population is *a *and a proportion *g *of each female's offspring are female. The basal death rate is *b*_0 _and the population's size is density-regulated in a linear manner according to a standard mass action assumption [53] with a density coefficient of *s *such that the total death rate is *b*_0_+*sH*. A population that harbours a single feminising parasite at prevalence *y *consists of *X_m _*uninfected males, *X_f _*uninfected females, *Y_m _*infected males and *Y_f _*infected females. A proportion *ε *of the offspring of infected females acquires the parasite by vertical transmission and a proportion *θ *of male offspring infected by vertical transmission are feminised while the remainder become infected males. Horizontal transmission is assumed to occur either through direct contact between infected and uninfected hosts or through the transmission of short-lived parasite spores between hosts via an environmental medium such as air, water or food. Following the methodology of Anderson & May (1981), the rate of horizontal transmission is considered to be directly proportional to the rate at which uninfected hosts encounter infected hosts and has the proportionality constant *β*. Encounters between males and females are unbiased and proportional to their density in the population. The net rate of horizontal transmission to uninfected male hosts is therefore *βX_m_*(*Y_m_+Y_f_*) and the net rate of horizontal transmission to female hosts is *βX_f_*(*Y_m_+Y_f_*). The parasite imposes a viability cost upon infected hosts such that the parasite-induced death rate is *α*. This viability cost applies equally to infected male hosts and infected female hosts. The rate at which infected hosts clear the parasite, regaining uninfected status, is *β*.

When two feminising parasites (parasite 1 and parasite 2) occur within the same population of hosts, the vertical transmission efficiency, feminisation efficiency, virulence, horizontal transmission efficiency and rate of clearance of parasite 1 are respectively defined as *ε_1_, θ_1_, α_1_, β_1 _*and *β_1_*. The number of infected males is *Y_m _*and the number of infected females is *Y_f_*. Parasite 2 has the equivalent parameters *ε_2_, θ_2_, α_2_, β_2 _*and *β_2 _*and infects *Z_m _*males and *Z_f _*females. If one parasite infects a host that is already infected with the other parasite, the invader displaces the resident (i.e. superinfection occurs) with probability *μ_1 _*(invading parasite 1 displaces resident parasite 2) or *μ_2 _*(invading parasite 2 displaces resident parasite 1). Hence the dynamics of a population infected with both parasites can be described by the following set of equations (1-7):

(1) $$\begin{array}{cc} \text{d}X_{f}/)dt=ag\left(X_{f}+Y_{f}\left(1-\varepsilon_{1}\right)+Z_{f}\left(1-\varepsilon_{2}\right)\right)\\ +Y_{f}\gamma_{1}+Z_{f}\gamma_{2}-X_{f}(b_{0}+sH+\beta_{1}\left(Y_{m}-Y_{f}\right) & \\ +\beta \left(Z_{m}+Z_{f}\right)) \end{array}$$

(2) $$\begin{array}{c} \text{d}X_{m}/)\text{d}t=\left(a-ag\right)\left(X_{f}+Y_{f}\left(1-\varepsilon_{1}\right)+Z_{f}\left(1-\varepsilon_{2}\right)\right)\\ +Y_{m}\gamma_{1}+Z_{m}\gamma_{2}-X_{m}(b_{0}+sH+\beta_{1}\left(Y_{m}+Y_{f}\right)\\ +\beta_{2}\left(Z_{m}+Z_{f}\right)) \end{array}$$

(3) $$\begin{array}{c} \text{d}Y_{m}/)\text{d}t=Y_{f}a\varepsilon_{1}\left(1-g-\theta_{1}+\theta_{1}g\right)\\ -Y_{m}\left(\alpha_{1}+\gamma_{1}+b_{0}+sH\right)\\ +\beta_{1}\left(X_{m}+\mu_{1}Z_{m}\right)\left(Y_{m}+Y_{f}\right)\\ -\beta_{2}\mu_{2}Y_{m}\left(Z_{m}+Z_{f}\right) \end{array}$$

(4) $$\begin{array}{c} \text{d}Y_{f}/)\text{d}t\text{ = }Y_{f}\text{a}\varepsilon_{\text{1}}\text{( }\theta_{\text{1}}\text{ + }g\text{ - }\theta_{\text{1}}g\text{)}\\ \text{ - }Y_{f}\text{ (}\alpha_{\text{1}}\text{ + }\gamma_{\text{1}}\text{ + }b_{\text{0}}\text{ + }sH\text{)}\\ \text{ + }\beta_{\text{1}}\text{ (}X_{f}\text{ + }\mu_{\text{1}}Z_{f}\text{)(}Y_{m}\text{ + }Y_{f}\text{)}\\ \text{ - }\beta_{\text{2}}\mu_{\text{2}}Y_{f}\text{(}Z_{m}\text{ + }Z_{f}\text{)} \end{array}$$

(5) $$\begin{array}{c} \text{d}Z_{m}/)\text{d}t=Z_{f}a\varepsilon_{2}\left(1-g-\theta_{2}+\theta_{2}g\right)\\ -Z_{m}\left(\alpha_{2}+\gamma_{2}+b_{0}+sH\right)\\ +\beta_{2}\left(X_{m}+\mu_{2}Y_{m}\right)\left(Z_{m}+Z_{f}\right)\\ -\beta_{1}\mu_{1}Z_{m}\left(Y_{m}+Y_{f}\right) \end{array}$$

(6) $$\begin{array}{c} \text{d}Z_{f}/)\text{d}t=Z_{f}a\varepsilon_{2}\left(\theta_{2}+g-\theta_{2}g\right)\\ -Z_{f}\left(\alpha_{2}+\gamma_{2}+b_{0}+sH\right)\\ +\beta_{2}\left(X_{f}+\mu_{2}Y_{f}\right)\left(Z_{m}+Z_{f}\right)\\ -\beta_{1}\mu_{1}Z_{f}\left(Y_{m}+Y_{f}\right) \end{array}$$

(7) $$\begin{array}{c} \text{d}H/)\text{d}t=a\left(X_{f}+Y_{f}+Z_{f}\right)-H\left(b_{0}+sH\right)\\ -\alpha_{1}\left(Y_{m}+Y_{f}\right)-\alpha_{2}\left(Z_{m}+Z_{f}\right) \end{array}$$

### Application of the model to *Nosema granulosis*

The model was instantiated using parameters estimated for the feminising microsporidian parasite *Nosema granulosis *in the crustacean host *Gammarus duebeni*. The parasite parameters (*ε *= 0.84, *θ *= 0.66, *α *= 0.001, *β *= 0, *γ *= 0) were derived from data published by Terry et al. [50] while the host parameters (*a *= 2, *b*_0 _= 0.12, *s *= 0.000042, *g *= 0.27, *K *= 10000) were derived from data published by Kelly et al. [54]. Competition between parasite strains that differed with regard to horizontal transmission and virulence but were otherwise similar was simulated by making a parasite's virulence *α *a linear function of its vertical transmission *ε *and its horizontal transmission *β *such that:

(8) α=λε+σβ

where *λ *and *σ *are constants, and allowing *β *to vary between strains.

This parameter set was used initially to investigate the sensitivity of an *N. granulosis*-like parasite's basic rate of increase to changes in the rate parameters *α, β, γ, ε *and *θ*. Each parameter was varied independently while the other parameters were held at default *N. granulosis *values. Parameter sets for mutant *N. granulosis*-like parasites, capable of horizontal transmission but with increased virulence were also generated. These were used to examine the impact of infection with feminizing parasites upon the host population's density and the effect of varying the level of superinfection upon the ability of two parasites to coexist. Finally, simulations were conducted in which an *N. granulosis*-like parasite without vertical transmission was allowed to attain equilibrium prevalence in host populations of sizes between 0 and 1000 individuals. A mutant was then introduced for which the virulence associated with vertical transmission (*σ*) varied between 0 and 300. For every value of *σ*, the level of horizontal transmission that provided the highest rate of increase (β*) was calculated. This allowed the model to be used to investigate conditions under which a population containing a solely vertically transmitted parasite could be invaded by a horizontally transmitted mutant.

## Results

### Host population dynamics

The carrying capacity *K *of the uninfected population occurs at the equilibrium point H* at which density-dependent mortality prevents further growth of the population such that:

(9) $$H^{*}=K\equiv \left(ag-b_{0}\right)∕s$$

The net rate of increase in the number of uninfected female hosts in the population (*R*_0_) is found by dividing the number of female offspring produced by a single uninfected female by the death rate of uninfected females in an uninfected population of size *H*. This gives the equation:

(10) $$R_{0}=\frac{ag}{b_{0}+sH}$$

### Single feminising parasite

A parasite lacking horizontal transmission is not transmitted through male hosts and so its basic reproductive rate (*R*_1_) is found by dividing the number of infected female offspring produced by a single infected female by the rate at which infected females are lost from the population due to natural mortality, parasite virulence or recovery. This gives the equation:

(11) $$R_{1}=\frac{a\varepsilon \left(\theta +g-\theta g\right)}{b_{0}+sH+\alpha +\gamma }$$

Invasion conditions for such a vertically transmitted feminising parasite are:

(12) $$R_{1}>R_{0}$$

That is, a feminising, vertically transmitted parasite can only invade a population of hosts if it causes the number of infected daughters produced by an infected female to exceed the number of uninfected daughters produced by an uninfected female [44,45,55]. If the host population is at equilibrium density then R_0 _= 1 and so inequality 12 becomes R_1 _> 1. This is equivalent to the conditions given for invasion and persistence of a horizontally transmitted parasite by Anderson and May [56] and of a parasite with horizontal and vertical transmission by Lipsitch et al. [57]. The maximum level of virulence possible if a vertically transmitted feminiser is to maintain its prevalence depends, crucially upon the sex ratio produced by uninfected hosts (*g*) as well as upon the parasite's rates of vertical transmission and feminisation [44,52,55].

The basic rate of increase of a feminising parasite with vertical and horizontal transmission (*R*_1_) is derived as

(13) $$R_{1}=\frac{\beta \left(X_{m}+X_{f}\right)+a\varepsilon \left(\theta +g-\theta g\right)}{b_{0}+sH+\alpha +\gamma }$$

and it can invade a population of uninfected hosts at equilibrium if *R*_1 _> 1. This condition specifies that a single infected individual in an otherwise uninfected population must give rise to at least one infected individual, following Lipsitch et al. [57]. The invasion success of the parasite with horizontal transmission therefore depends not only upon the sex ratio produced by uninfected hosts but also upon the availability of uninfected hosts and therefore initially by the density of the host population. If a parasite is unable to invade through vertical transmission alone (i.e. *R*_1 _as defined in equation 11 is less than one) then horizontal transmission will only allow it to invade if

(14) $$\beta >\frac{b_{0}+sH+\alpha +\gamma +a\varepsilon \left(\theta +g-\theta g\right)}{X_{m}+X_{f}}$$

Horizontal transmission depends upon the production of infective stages, a process that depletes the host's energy resources and often causes damage to host tissues. High rates of horizontal transmission are therefore predicted to reduce the lifetime reproductive success of infected hosts and hence to reduce the efficiency of vertical transmission. If vertical transmission and horizontal transmission both impose linear viability costs and these costs are additive then:

(15) α=λε+σβ

where the constant *λ *defines the slope of the increase in virulence with vertical transmission and *σ *defines the slope of the increase in virulence with horizontal transmission, then horizontal transmission only increases the capacity of a parasite to invade a host population if:

(16) $$\sigma <X_{m}+X_{f}$$

The maximum virulence at which a parasite is able to invade by horizontal transmission therefore depends upon the density of uninfected hosts in the population. The greater the cost of horizontal transmission, the higher the host density must be in order for the parasite to benefit from horizontal transmission.

### Two feminising parasites

Competitive exclusion of one parasite by another has been demonstrated analytically in simpler models where both parasites are solely vertically transmitted (*β*_1 _= *β*_2 _= 0) [44] or both parasites are solely horizontally transmitted (*ε*_1 _= *ε*_2 _= 0) [56]. Simulations performed using the current model under the conditions *β*_1 _= *β*_2 _= 0 or *ε*_1 _= *ε*_2 _= 0 confirmed these results. However, for cases in which one or both parasites are capable of both vertical and horizontal transmission, simulations often indicated stable coexistance of two feminising parasites under conditions in which the parasite with the most effective vertical transmission had the least effective horizontal transmission. Under these conditions, the spread of one parasite is limited by the host's sex ratio while the spread of the other parasite is limited by the host's population density. This finding accords with the convention that as long as two competitors are limited by different factors, coexistence is possible [58].

**Figure 1:**
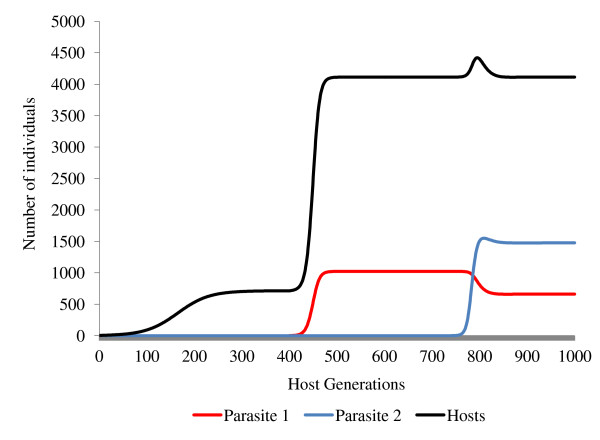
**Response of a host population infected with two feminising parasites to changes in vertical transmission efficiency (*ε*)**. Effects upon equilibrium host population size (H*), number of hosts infected with parasite 1 (Y*) and number of hosts infected with parasite 2 (Z*) of varying the vertical transmission efficiency (*ε*_2_) of parasite 2. The host population has parameters (*a = *2, *g = *0.27, *b*0 = 0.12, *s *= 0.000042, *R*_0 _= 2.06). Default parameters for parasite 1 are (*ε = *0.84, *θ = *0.66, *α = *0.10, *β = *0, *γ = *0, *μ *= 0, *R_1 _*= 4.80), while for parasite 2 they are (*ε = *0.84, *θ = *0.66, *α = *0.045, *β = *0.0002, *γ = *0, *μ *= 0, *R_2 _= *4.26). With all rate parameters set at default values, the two parasites coexist stably.

A simulation in which two parasites achieved stable coexistence within 1000 host generations is presented in Figure 1. In order for these conditions to be met, the parasite with the lowest reproductive rate in an uninfected host population must have a reproductive rate greater than 1 in a population in which its competitor occurs at equilibrium prevalence. These conditions are likely to occur when the first parasite is a feminiser with superior vertical transmission and the second parasite has superior horizontal transmission. This is because the presence of a feminising parasite can increase the host population's growth rate and hence its equilibrium density.

**Figure 2:**
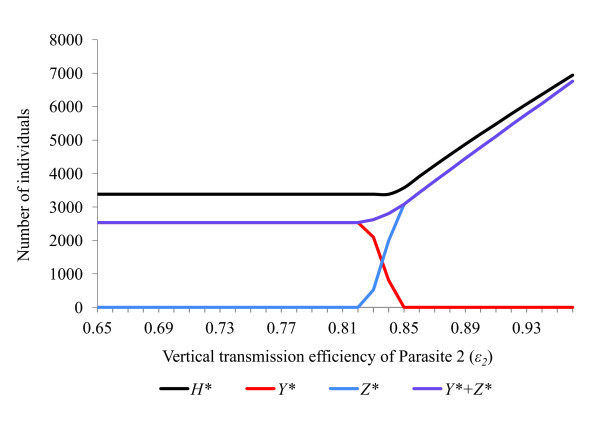
**Response of a host population infected with two feminising parasites to changes in virulence (*α*)**. Effects upon equilibrium host population size (H*), number of hosts infected with parasite 1 (Y*) and number of hosts infected with parasite 2 (Z*) of varying the virulence (*α*_2_) of parasite 2. The host population has parameters (*a = *2, *g = *0.27, *b*0 = 0.12, *s *= 0.000042, *R*_0 _= 2.06). Default parameters for parasite 1 are (*ε = *0.84, *θ = *0.66, *α = *0.10, *β = *0, *γ = *0, *μ *= 0, *R_1 _*= 4.80), while for parasite 2 they are (*ε = *0.84, *θ = *0.66, *α = *0.045, *β = *0.0002, *γ = *0, *μ *= 0, *R_2 _= *4.26). With all rate parameters set at default values, the two parasites coexist stably.

**Figure 3:**
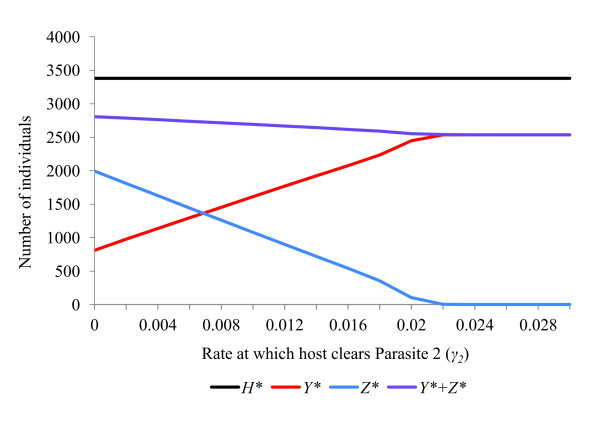
**Response of a host population infected with two feminising parasites to changes in recovery rate (*γ*)**. Effects upon equilibrium host population size (H*), number of hosts infected with parasite 1 (Y*) and number of hosts infected with parasite 2 (Z*) of varying the recovery rate (*γ*_2_) of parasite 2. The host population has parameters (*a = *2, *g = *0.27, *b*0 = 0.12, *s *= 0.000042, *R*_0 _= 2.06). Default parameters for parasite 1 are (*ε = *0.84, *θ = *0.66, *α = *0.10, *β = *0, *γ = *0, *μ *= 0, *R_1 _*= 4.80), while for parasite 2 they are (*ε = *0.84, *θ = *0.66, *α = *0.045, *β = *0.0002, *γ = *0, *μ *= 0, *R_2 _= *4.26). With all rate parameters set at default values, the two parasites coexist stably.

**Figure 4:**
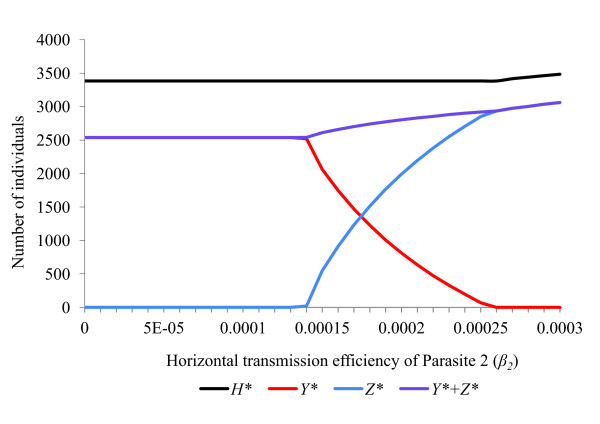
**Response of a host population infected with two feminising parasites to changes in feminisation efficiency (*θ*)**. Effects upon equilibrium host population size (H*), number of hosts infected with parasite 1 (Y*) and number of hosts infected with parasite 2 (Z*) of varying the feminisation efficiency (*θ*_2_) of parasite 2. The host population has parameters (*a = *2, *g = *0.27, *b*0 = 0.12, *s *= 0.000042, *R*_0 _= 2.06). Default parameters for parasite 1 are (*ε = *0.84, *θ = *0.66, *α = *0.10, *β = *0, *γ = *0, *μ *= 0, *R_1 _*= 4.80), while for parasite 2 they are (*ε = *0.84, *θ = *0.66, *α = *0.045, *β = *0.0002, *γ = *0, *μ *= 0, *R_2 _= *4.26). With all rate parameters set at default values, the two parasites coexist stably.

**Figure 5:**
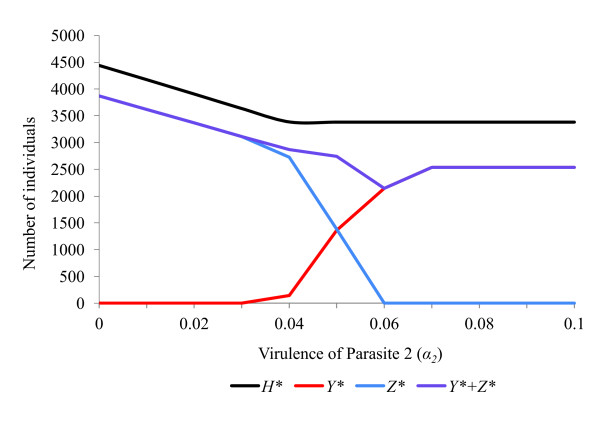
**Response of a host population infected with two feminising parasites to changes in *horizontal transmission efficiency *(*β*)**. Effects upon equilibrium host population size (H*), number of hosts infected with parasite 1 (Y*) and number of hosts infected with parasite 2 (Z*) of varying the horizontal transmission efficiency (*β*_2_) of parasite 2. The host population has parameters (*a = *2, *g = *0.27, *b*0 = 0.12, *s *= 0.000042, *R*_0 _= 2.06). Default parameters for parasite 1 are (*ε = *0.84, *θ = *0.66, *α = *0.10, *β = *0, *γ = *0, *μ *= 0, *R_1 _*= 4.80), while for parasite 2 they are (*ε = *0.84, *θ = *0.66, *α = *0.045, *β = *0.0002, *γ = *0, *μ *= 0, *R_2 _= *4.26). With all rate parameters set at default values, the two parasites coexist stably.

**Figure 6:**
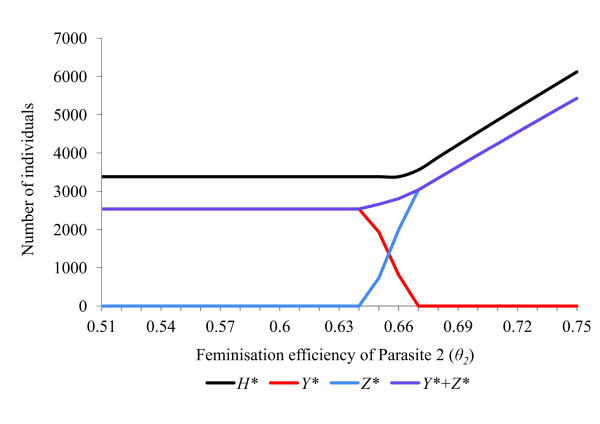
**A simulation demonstrating the interaction of two feminising parasites and their joint effects upon the density of a host population**. Parasite 2 is capable of both vertical and horizontal transmission (*ε = *0.7, *θ = *0.2, *α = *0.02, *β = *0.0002) but is unable to invade a host population (*a = *2.3, *g = *0.5, *b*_0 _= 0.12, *s *= 0.000042) at its uninfected equilibrium size of H* = 714. Parasite 1 is incapable of horizontal transmission but has more efficient vertical transmission and feminisation (*ε = *0.75, *θ = *0.5, *α = *0.001, *β = *0) and so is able to invade an uninfected population. Addition of a single female infected with parasite 1 at generation 400 results in the rapid spread of parasite 1 to equilibrium prevalence (y* = 0.25), increasing the equilibrium population size (H*) to 4113. This increase in population size allows parasite 2 to invade and achieve a prevalence of 0.36, driving the prevalence of parasite 1 down to 0.16.

The rate parameters *α, β, γ, θ *and *ε *all affect the ability of a feminising parasite to invade, persist and compete with other parasites within a host population (Figures 2, 3, 4, 5, 6). These factors act both through their direct effects upon the parasite's basic rate of increase, and indirectly, by altering the equilibrium density of the host population. Any increase in the basic rate of increase of a feminising parasite will tend to increase the equilibrium density of the host population due to the increase in the proportion of female hosts.

In addition to increasing a parasite's rate of vertical transmission, feminisation has the potential to boost a parasite's rate of horizontal transmission. This is due to the increase in the equilibrium density of susceptible hosts in a population caused by the greater proportion of female hosts produced by feminisation. A net increase in the host's population density occurs whenever the increase in the population's rate of growth caused by feminisation exceeds the depressing effect of a parasite's virulence (i.e. where inequality 11 is true). If feminisation boosts the host's rate of increase, it thereby boosts horizontal transmission by increasing the density of susceptible hosts. Furthermore, in addition to enhancing its own horizontal transmission, the increase in the host's population density caused by a feminising parasite has the potential to boost the reproductive rate of any horizontally transmitted parasite that occurs within the same population. This can occur whenever the density of susceptible hosts present in the population when the feminising parasite is at equilibrium prevalence exceeds the density of susceptible hosts when the feminising parasite is absent. This occurs when:

(17) $$H^{*}-y^{*}H^{*}\left(1-\mu_{2}\right)>K$$

This effect can be seen in Figure 6, based on the two-parasite model (Equations 1-7) instantiated with data from the feminizing microsporidian *Nosema granulosis*. This illustrates the effect that changes in host population size can have upon the outcome of competition between horizontally and vertically transmitted parasite strains. By increasing the host's population density, a vertically transmitted feminising parasite can facilitate the invasion of a horizontally transmitted parasite. In cases in which two parasites coexist, the presence of a feminiser also allows a horizontally transmitted parasite to evolve higher virulence.

**Figure 7:**
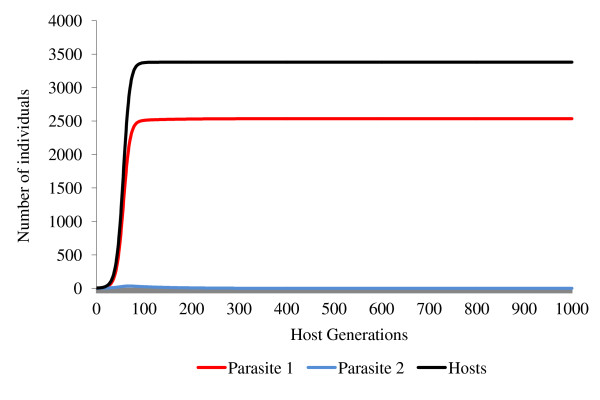
**Co-infection of two feminising parasites without superinfection**. Both parasites have the *N. granulosis *parameters *ε *= 0.84 and *θ *= 0.66 while the host has the *G. duebeni *parameters *a = *2, *g = *0.27, *b*_0 _= 0.12 and *s *= 0.000042. Parasite 1 is the ancestral *N. granulosis *strain with *α *= 0.001 and *β *= 0, while parasite 2 is a mutant, horizontally transmitted strain with *α *= 0.08 and *β *= 0.0002. When parasite 2 is incapable of superinfection (*μ *= 0), it is excluded by parasite 1.

**Figure 8:**
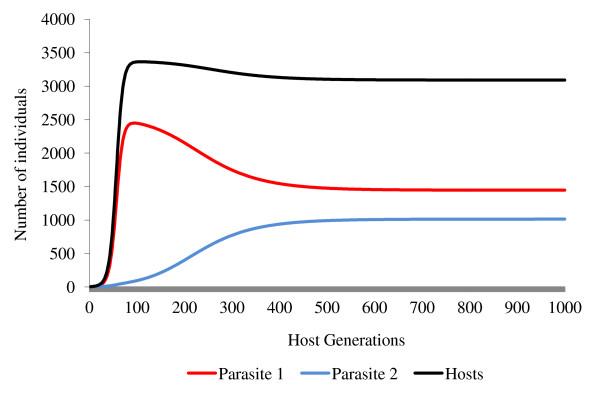
**Co-infection of two feminising parasites with low levels of superinfection**. Both parasites have the *N. granulosis *parameters *ε *= 0.84 and *θ *= 0.66 while the host has the *G. duebeni *parameters *a = *2, *g = *0.27, *b*_0 _= 0.12 and *s *= 0.000042. Parasite 1 is the ancestral *N. granulosis *strain with *α *= 0.001 and *β *= 0, while parasite 2 is a mutant, horizontally transmitted strain with *α *= 0.08 and *β *= 0.0002. When parasite 2 is capable of low levels of superinfection (*μ *= 0.06), it coexists with parasite 1.

**Figure 9:**
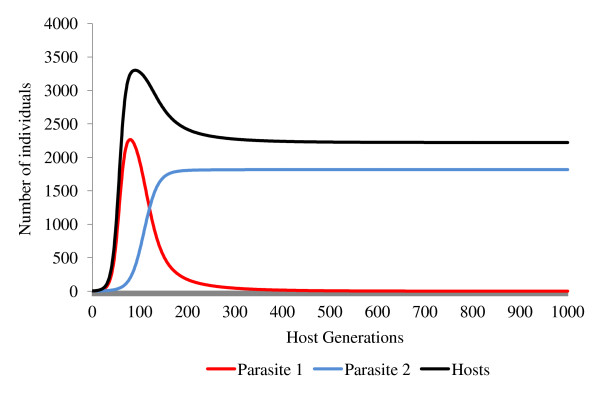
**Co-infection of two feminising parasites with high levels of superinfection**. Both parasites have the *N. granulosis *parameters *ε *= 0.84 and *θ *= 0.66 while the host has the *G. duebeni *parameters *a = *2, *g = *0.27, *b*_0 _= 0.12 and *s *= 0.000042. Parasite 1 is the ancestral *N. granulosis *strain with *α *= 0.001 and *β *= 0, while parasite 2 is a mutant, horizontally transmitted strain with *α *= 0.08 and *β *= 0.0002. When parasite 2 is capable of high levels of superinfection (*μ *= 0.16), parasite 2 excludes parasite 1

Where horizontal transmission occurs, superinfection can also play an important role in determining the outcome of competitive interactions between two feminising parasites (Figures 7, 8, 9). Unless the second parasite to invade a population is very effective at displacing its rival through superinfection (*μ_2 _*→1), its ability to invade a population by horizontal transmission is less than when it is the only parasite in the population. This is because a proportion of the population's female hosts have already been infected by the resident parasite and are therefore less susceptible to infection. The ability of a parasite to benefit from superinfection depends upon the efficiency of its horizontal transmission and so high levels of superinfection will generally favour parasites with high levels of horizontal transmission and hence with high levels of virulence.

### Evolution of transmission strategies

**Figure 10:**
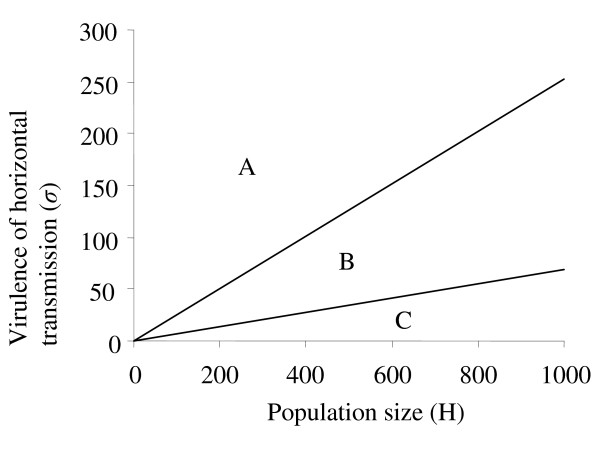
**Threshold coefficient of virulence (*σ*) and host population size (*H*) required for a horizontally transmitted mutant (parasite 2) to invade a population of vertically transmitted feminising parasites (parasite 1) at equilibrium prevalence**. In zone A, parasite 1 excludes parasite 2 and so the optimal level of horizontal transmission (β*) is 0. In zone B, parasites 1 and 2 coexist stably when horizontal transmission of parasite 2 = β*. In zone C, parasite 2 invades and displaces parasite 1 when horizontal transmission of parasite 2 = β*. Parameters for parasite 1 are estimated from *Nosema granulosis *infecting *Gammarus duebeni*: *ε *= 0.84, *θ *= 0.66, *g *= 0.27, *a = *2, *β *= 0, *b*_0 _*= *0.12, *s *= 0.000042, y* = 0.747. Parameters for parasite 2 are identical to those of parasite 1 except that *β *> 0.

In small host populations, the model predicts that a solely vertically transmitted feminizing parasite can exclude any horizontally transmitted mutant as long as there is a significant cost to horizontal transmission in terms of virulence (Figure 10). Within this parameter region, β* = 0 and the basic rate of increase of the mutant (*R*_2_) is less than one for all non-zero values of *β*. In large populations where the virulence cost of horizontal transmission is low, a narrow zone exists in which a horizontally transmitted mutant can exclude the original, solely vertically transmitted parasite. For parameters within this zone, β* > 0 and *R_2 _*> 1 when the original parasite occurs at equilibrium prevalence (y*). However, the basic rate of increase of the solely vertically transmitted parasite (*R*_1_) is less than one when the mutant is at equilibrium prevalence (z*). Finally, a sizeable intermediate parameter region exists within which β* > 1 and *R*_2 _> 1 at y* but *R*_1 _> 1at z*. Simulations indicate that, within this zone, coexistence of solely vertically transmitted feminisers with horizontally transmitted mutants can occur.

## Discussion

Our model indicates that low levels of horizontal transmission can benefit a feminising parasite by increasing its ability to displace a rival parasite or by allowing it to coexist with another parasite that has superior vertical transmission. These benefits accrue even when horizontal transmission is costly relative to vertical transmission. Unlike the models of Engelstadter and Hurst [47] and Yamauchi et al [48] in which horizontal transmission occurs only via adult males, our model allows horizontal transmission to occur through both male and female hosts. This alters the trade-off between horizontal and vertical transmission. Feminised hosts can still transmit the parasite horizontally as well as vertically and so feminisation and horizontal transmission are not mutually exclusive strategies. However, the additional virulence caused by horizontal transmission reduces the capacity of female hosts to transmit the parasite vertically, reducing the efficiency of feminisation, so a tradeoff still exists.

Because horizontal transmission is limited by the host's population density and feminisation is limited by the host's sex ratio, horizontal transmission by at least one of a pair of feminising parasites can allow the two parasites to coexist, even when one parasite has less efficient vertical transmission than the other. Cryptic horizontal transmission can therefore explain the numerous cases in which several SRD parasites have been found to coexist, without the need to invoke complex systems of virulence and resistance genes.

The probability of coexistence can be increased still further because a feminising parasite has the potential to boost the host population's rate of increase, mitigating or even reversing the depressing effect of parasite virulence on host population density. The feminising parasite is able to achieve this by mitigating the reduction in the host population's productivity due to the production of males (the "two fold cost of sex") [59]. By eliminating males, the feminiser frees resources that can then be harnessed for host population growth or stolen by other parasites. This potential for sex-ratio distorting parasites to increase the growth rate of host populations was predicted by Werren and Beukeboom [60] and by Hatcher et al. [61] and is included as an assumption in the model of Jones et al. [49]. This phenomenon is an emergent property of our model and confirms the prediction of Jones at al. [49] that a feminizing parasite can increase the transmission and hence the optimum virulence of a horizontally transmitted parasite by increasing the host's population density. Our model also makes the prediction that a parasite using both vertical and horizontal transmission can potentially increase its horizontal transmission, as well as its vertical transmission, through feminization.

Jones et al. [49] do not model the sex ratio of the host population explicitly, effectively assuming that horizontal transmission occurs only through females and that the only effect of feminization is to increase the population's birth rate. They also appear to ignore the contribution of males to density regulation of the host population. Given that feminisation reduces the male birth rate as well as increasing the female birth rate, this omission may lead to underestimation of the impact of feminization upon host population density. In contrast, our model assumes that horizontal transmission occurs through both males and females and that both sexes contribute equally to density-regulation of the population size. Even this assumption may be overly conservative in some cases. In *G. duebeni*, for example, males are considerably larger than females and actively predate upon females and juveniles of their own species [62]. Hence, males are likely to have a greater regulatory effect upon *G. duebeni *population size than do females and the effect of feminization upon population size may be even greater than predicted in our model.

Although increasing the proportion of females in a population has the potential to increase the population's rate of increase, a minimum number of males (or rather, a minimum number of sperm) is required if the host population is to survive at all. McCauley & Taylor [63] and Hatcher et al. [61,64] both suggested that sex ratio distorters could depress infected host populations by skewing the sex ratio to the point at which the availability of sperm limited the population's rate of increase. Evidence for sperm-limitation exists in populations of *Acraea encedon *infected with male-killing *Wolbachia *[65] and males of the feminising parasite hosts *Armadillidium vulgare *and *Gammarus duebeni *also suffer from sperm depletion after multiple matings [66,67] suggesting a theoretical cost of sperm-limitation to infected populations. However, evidence of sperm-limitation is lacking in natural populations. Indeed, populations of *G. duebeni *on the island of Great Cumbrae, known to harbour the feminisers *N. granulosis *and *D. duebenum *at high prevalence [42], have male-biased sex ratios [54].

Judging by the predictions of our model, we consider it likely that parasites utilising both SRD and horizontal transmission are more common than is suggested by the scarce empirical evidence that has been produced so far. However, if horizontally transmitted SRD parasites are actually rare, this suggests that the trade-off between horizontal and vertical transmission must be extremely steep. If the production of many spores causes little more damage to the host than the production of few or, conversely, if the host tolerates a low parasite load with few ill effects but is crippled by a slightly higher load then the relationship between transmission and virulence may be asymptotic or sigmoid rather than linear. It is also considered likely that parasite transmission will saturate before virulence (Ebert 1998). This would alter the parameters under which horizontal transmission became advantageous and might also alter the parameters under which two parasite were able to coexist. Finally, there may be costs associated with feminisation. These might include virulence arising from the need for parasite stages to infect specific tissues or to attain greater numbers than would otherwise be optimal for vertical transmission. An additional cost of feminisation occurs for microsporidian parasites of amphipod crustaceans such as *Gammarus duebeni *and *Echinogammarus marinus*, in which infected hosts suffer an increased risk of intersexuality [68,69].

A possible candidate for measuring the separate virulence costs of horizontal transmission, vertical transmission and feminisation is the microsporidian *Dictyocoela duebenum *in *G. duebeni*. This parasite appears to cause two different forms of pathology. One is asymptomatic and associated with vertical transmission and feminisation [42] while the other results in replacement of muscle tissue with masses of spores, and is presumably associated with horizontal transmission [33]. By examining hosts exhibiting these two forms of pathology, the relative virulence costs of horizontal transmission and feminisation could be compared for the same parasite strain.

## Conclusions

On the basis of a simulations involving two feminising parasites in a single host population, the following predictions are made:

Horizontal transmission is advantageous to feminising parasites under a wide range of conditions.

Horizontal transmission facilitates coexistence of multiple feminising parasites within a single host population

The presence of a feminising parasite can increase host population density, hence creating conditions under which a mutant or another parasite species with greater horizontal transmission and/or higher virulence can invade.

## Authors' contributions

JEI constructed the mathematical model and drafted the manuscript. MJH assisted with construction of the model and helped to draft the manuscript. JES and AMD conceived of the study, participated in its design and coordination and helped to draft the manuscript. All authors read and approved the final manuscript.
